# Beyond the light-driven pacemaker: the suprachiasmatic nucleus as a dynamic coordinator of environmental cues

**DOI:** 10.3389/fnins.2026.1937340

**Published:** 2026-10-09

**Authors:** Tao Zhang, Kai Huang, Jing Yu, Shaojun Wu, Qiaocheng Zhai

**Affiliations:** 1 Jiangsu Province Engineering Research Center of Development and Translation of Key Technologies for Chronic Disease Prevention and Control, Suzhou Vocational Health College, Suzhou, Jiangsu, China; 2 Orthopaedic Institute, Wuxi Ninth People's Hospital Affiliated to Soochow University, Wuxi, China; 3 Peking University-Nanjing Institute of Translational Medicine, Nanjing, China; 4 College of Pharmacy, Suzhou Vocational Health College, Suzhou, China; 5 Core Facility, The Quzhou Affiliated Hospital of Wenzhou Medical University, Quzhou People's Hospital, Quzhou, Zhejiang, China

**Keywords:** circadian clock, light signal, neuronal heterogeneity, suprachiasmatic nucleus, time-restricted feeding

## Abstract

The suprachiasmatic nucleus (SCN) is the central mammalian circadian pacemaker, but its function extends beyond passive entrainment to light. Here, we synthesize evidence that the SCN is a heterogeneous and plastic network that integrates photic, metabolic, astrocytic, and thermal information across daily entrainment, seasonal adaptation, jet lag, and chronic circadian misalignment. VIP and GRP neurons provide privileged access for retinal input and network synchronization, AVP neurons support intrinsic rhythmicity and output, and the broad *Nms*-expressing ensemble and CCK-defined subtypes contribute to arousal and adaptive network plasticity. We propose an integrative circuit model in which retinal signals preferentially engage core VIP/GRP neurons, whereas feeding and metabolic information influences VIP and AVP populations through circulating factors, the dorsomedial hypothalamus, the intergeniculate leaflet, and metabolically responsive astrocytes. Rhythmic astrocytic GABA and glutamate release, together with VIP-AVP coupling and overlapping VIP^+^/NMS^+^ and AVP^+^/NMS^+^ subpopulations, may coordinate phase resetting and sleep-wake rhythms. The timing mechanism underlying food anticipatory activity (FAA) is attributed primarily to distributed extra-SCN food-entrainable circuits, whereas the SCN may gate or modulate its behavioral expression rather than generate it autonomously. Light remains the dominant zeitgeber. Under standard light-dark conditions, time-restricted feeding more commonly modulates SCN neuronal activity, calcium dynamics, neuropeptide output, and behavior than resets the core TTFL; molecular resetting has been reported only under specific genetic, temporal, dietary, lighting, or network conditions. When light and feeding cues conflict, rapid ventral resetting and slower dorsal reentrainment generate transient intranuclear and systemic desynchrony rather than simple dominance by either cue. Biological sex and aging further shape this integrative capacity, while temperature compensation delineates the boundary between resistance to routine thermal fluctuations and conditional responses to stronger thermal challenges. This framework helps explain circadian disruption in jet lag, shift work, social jetlag, and metabolic disease, and supports circadian medicine strategies that align timed light, feeding, exercise, sleep, and pharmacotherapy across central and peripheral clocks.

## 1 Introduction

The daily rotation of our planet imposes a fundamental 24-h rhythm on the environment, a challenge that has driven the evolution of endogenous circadian clock systems across nearly all forms of life, from fungi and plants to mammals, including humans (Dallmann et al., 2014). These intrinsic timekeeping systems generate ~24-h oscillations that regulate a vast array of physiological and behavioral processes, including the sleep-wake cycle, locomotor activity, and hormone secretion. In mammals, the suprachiasmatic nucleus (SCN) of the ventral hypothalamus is unequivocally established as the central regulator of this circadian hierarchy. Pioneering lesion studies demonstrated that SCN ablation permanently disrupts circadian rhythms in behaviors such as wheel-running and in hormonal release (Stephan and Zucker, 1972; Moore and Eichler, 1972), while subsequent transplantation experiments confirmed the SCN as the master circadian pacemaker, capable of restoring rhythmicity to arrhythmic hosts (Weaver, 1998; Ralph et al., 1990).

The molecular gears of this biological clock were brought to light by the groundbreaking work of Hall, Rosbash, and Young, recognized by the 2017 Nobel Prize. Their work defined the core transcriptional-translational feedback loop (TTFL), wherein the BMAL1 and CLOCK heterodimer activates the transcription of Period (*Per*) and Cryptochrome (*Cry*) genes. The resulting PER and CRY proteins then accumulate, translocate to the nucleus, and inhibit their own transcription, creating a self-sustaining cycle with a period of approximately 24 h (Cox and Takahashi, 2019). An auxiliary feedback loop involving REV-ERB and RORα provides stability and robustness to this core oscillator (Cox and Takahashi, 2019). This cell-autonomous TTFL mechanism operates not only within the neurons of the SCN but in virtually all nucleated cells throughout the body.

As the central pacemaker, the SCN's critical function extends beyond generating an intrinsic rhythm to detecting and integrating a variety of environmental time cues, known as “zeitgebers” (Schulz and Steimer, 2009; Gabriel and Zierath, 2019; Gillman et al., 2019). The most potent of these is light. Photic information is captured by intrinsically photosensitive retinal ganglion cells (ipRGCs) and relayed directly to the ventral SCN via the retinohypothalamic tract (RHT) (LeGates et al., 2014). This light signal, mediated by neurotransmitters like glutamate and PACAP, resets the SCN clock daily to ensure alignment with the external solar day (Fernandez et al., 2016; Morin, 1994; Michel et al., 2006; Meijer et al., 1993; Ebling, 1996). The SCN, in turn, broadcasts this synchronized time signal, coordinating circadian rhythms in other brain regions through direct neural projections (Kalsbeek et al., 2006; Gizowski et al., 2016; Colwell, 2011) and synchronizing peripheral tissue clocks via autonomic nervous system outputs and rhythmic hormone secretion, such as glucocorticoids (Morf and Schibler, 2013; Gerber et al., 2013; Hastings et al., 2003; Mohawk et al., 2012).

However, the environment presents multiple, sometimes competing, time cues. While light is the primary zeitgeber, the SCN must also process salient non-photic signals, chief among them being feeding rhythms and energy status. The classical view of the SCN as a rigid, light-dominated pacemaker is therefore giving way to a more nuanced paradigm: the SCN as a context-dependent coordinator of multiple zeitgebers. This modern perspective posits that the SCN actively weighs and reconciles photic inputs with non-photic cues to produce adaptive physiological and behavioral outputs, a computational feat enabled by its remarkable cellular and network complexity.

In this review, we will systematically explore this integrative capacity. We will first deconstruct the SCN's heterogeneous cellular composition, detailing how specialized neuronal subtypes, including those expressing vasoactive intestinal peptide (VIP), arginine vasopressin (AVP), gastrin-releasing peptide (GRP), neuromedin S (NMS), and cholecystokinin (CCK), form a sophisticated neural circuit for signal processing. We will then examine the mechanisms by which this circuit encodes temporal information across different contexts, from daily light stimulation and seasonal photoperiod changes to the disruptive state of jet lag. Finally, we will challenge the traditional hierarchy of zeitgebers by evaluating the growing body of evidence demonstrating the SCN's sensitivity to dietary cues, with a particular focus on the effects of time-restricted feeding. By synthesizing this knowledge, we aim to reframe the SCN as a plastic and adaptive hub, thereby clarifying its central role in environmental timekeeping and highlighting new, translationally relevant directions for future research.

Throughout this review, we distinguish SCN responsiveness from molecular-clock resetting. Acute changes in c-FOS, neuronal firing, calcium dynamics, neuropeptide expression, or behavioral output are treated as cellular, network, or output modulation, whereas resetting or entrainment refers to a sustained phase change in rhythmic core TTFL markers, ideally demonstrated under constant conditions or persisting after withdrawal of the feeding cue.

A related conceptual distinction concerns convergence and functional integration. In this review, we define functional integration as a demonstrable interaction in which the presence, phase, or strength of one zeitgeber changes the response to another relative to the responses elicited by each cue alone. Such integration may occur at a specific cellular, network, molecular, or behavioral level and does not imply that all inputs are fused into a single signal or that integration at one level extends to others. Anatomical co-access or parallel responses alone indicate potential convergence rather than functional integration. Accordingly, a central question of this review is how VIP, GRP, AVP, NMS, and CCK populations interact when photic and feeding cues coincide or conflict.

## 2 The SCN as a heterogeneous neural network: neuropeptide-defined ensembles and their behavioral regulation

Anatomically, the SCN is a compact but highly heterogeneous nucleus (~400–600 μm in diameter) comprising approximately 20,000 neurons (Abrahamson and Moore, 2001; Van den Pol, 1980). This cellular diversity is reflected in the expression of hundreds of neuropeptides (Lee et al., 2013, 2010) and highly varied patterns of neuronal activity across multiple spatiotemporal scales, as revealed by advanced techniques like two-photon calcium imaging (Wang et al., 2024). Neuropeptide expression in the SCN is combinatorial and should not be interpreted as defining mutually exclusive neuronal classes. Although VIP and AVP predominantly mark core- and shell-associated ensembles, respectively, both comprise molecularly heterogeneous subtypes that co-express additional neuropeptides. Nms is a particularly broad, cross-cutting marker: *Nms*-expressing neurons constitute approximately 40% of mouse SCN neurons and encompass most, but not all, VIP^+^ and AVP^+^ neurons (Lee et al., 2015). Single-cell transcriptomic studies accordingly identify *Vip*^+^/*Nms*^+^ and *Avp*^+^/*Nms*^+^ subtypes rather than a single homogeneous NMS population (Wen et al., 2020). Throughout this review, neuropeptide labels therefore refer to partially overlapping, marker-defined ensembles unless a transcriptionally or anatomically segregated subtype is explicitly specified. Advances in proteomics and single-cell RNA sequencing are progressively refining this complex cellular taxonomy and clarifying how these overlapping ensembles collectively contribute to SCN network dynamics and behavioral outputs (Park et al., 2016; Wen et al., 2020). Figure 1 provides a schematic overview of the spatial and functional relationships among major SCN neuropeptide-defined ensembles, whereas Table 1 summarizes their anatomical distributions and overlaps, environmental responsiveness, principal network roles, and reported physiological and behavioral associations.

**Figure 1:**
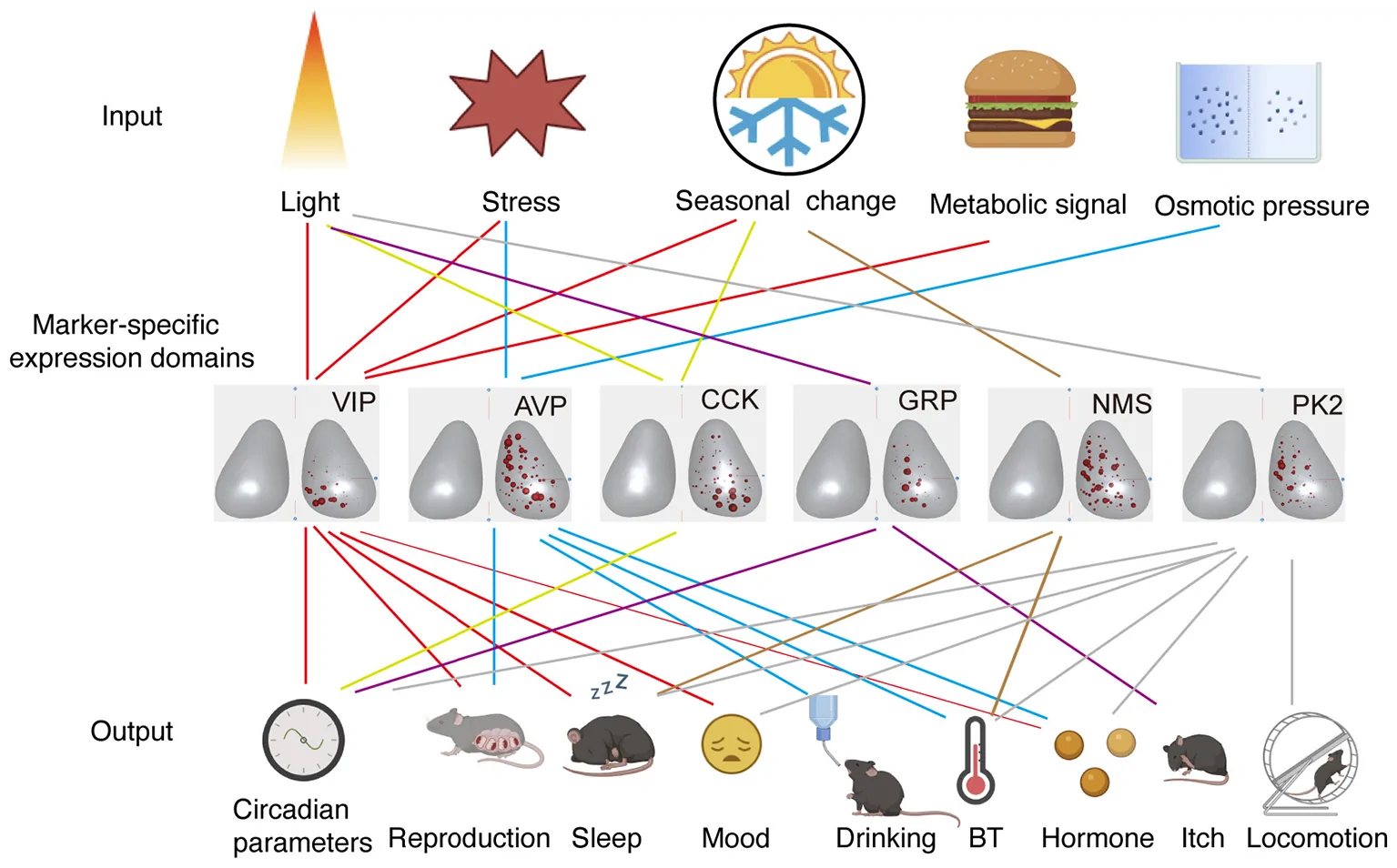
Major neuropeptide expression domains, their distribution, responses to environmental signals, and regulated physiological functions. Distribution of major neuropeptide expression domains in the SCN (red bubbles; data from http://yanlab.org.cn/scn-atlas/). Different SCN domains respond to varying environmental signals, including light stimulation, stress, seasonal changes, metabolic signals, and osmotic pressure. Downstream physiological activities regulated by different neuron types include circadian parameters (changes in neuronal activity or the TTFL can cause changes in period, phase shifts, or arrhythmia), reproduction, sleep, mood, drinking, body temperature (BT), hormones, itch, and locomotion. Input and output signals for each specific domain are indicated by colored lines. The neuropeptide markers shown here define partially overlapping expression domains rather than mutually exclusive neuronal populations. In particular, Nms expression spans the SCN core and shell and overlaps extensively with VIP^+^ and AVP^+^ neurons. The indicated functions summarize findings from marker-targeted experiments and should not be interpreted as functions unique to a single peptide or non-overlapping cell class.

**Table 1:** Anatomical organization, environmental responsiveness, and reported functions of major neuropeptide-defined ensembles and signaling domains in the suprachiasmatic nucleus.

Marker-defined ensemble or signaling domain	Approximate SCN distribution and overlap	Environmental responsiveness or modulatory context	Principal circadian and network roles	Reported physiological or behavioral associations
VIP^+^ ensemble	Predominantly ventral/core; approximately 24% of SCN neurons. Partially overlaps with GRP^+^ cells and with the broad *Nms*-expressing ensemble.	Directly retinorecipient and strongly light responsive; also modulated by stress and metabolic state, including high-fat diet.	Major conduit for photic entrainment; promotes intercellular coupling, network synchrony, and phase resetting. Its network effects are partly separable from the cell-autonomous VIP-neuron clock.	Sleep promotion; locomotor rhythmicity; estrous-cycle and reproductive regulation; mood-related behavior.
GRP^+^ ensemble	Predominantly core localized; approximately 10% of SCN neurons. A subset co-expresses VIP.	Receives mainly intra-SCN input but also responds to light and visual cues, as indicated by acute clock-gene, immediate-early-gene, and calcium responses.	Supports photic phase resetting and network synchronization, particularly when VIP signaling is compromised; it is not essential for baseline rhythmicity or entrainment.	Circadian phase regulation; socially contagious itch and mimicry scratching; associated stress responses through PVT/PVN relays.
AVP^+^ ensemble	Predominantly dorsal shell; approximately 10%−30% of SCN neurons. Extensively overlaps with the broad *Nms*-expressing ensemble.	Responsive to stress-associated and osmotic signals and participates in intra-SCN interactions with light-responsive VIP^+^ cells.	Displays robust intrinsic rhythmicity; provides a TTFL-controlled rhythmic output; supports autonomous SCN oscillation and stabilizes ensemble phase.	Corticosterone and reproductive-hormone rhythms; reproductive timing; pre-sleep drinking; body-temperature regulation.
*Nms*-expressing ensemble	Broad, heterogeneous ensemble comprising approximately 40% of mouse SCN neurons and spanning core and shell. It includes most, but not all, VIP^+^ and AVP^+^ neurons, together with additional NMS^+^/VIP-/AVP-cells.	Shows photoperiod-dependent changes in cell abundance and projections. Rhythmic primary-cilium-mediated Sonic Hedgehog signaling provides an additional modulatory mechanism; a universal direct sensory-input route has not been established.	The *Nms*-defined cellular ensemble contributes to SCN synchrony and behavioral rhythmicity. Effects of *Nms*-Cre manipulations do not establish an indispensable role for the NMS peptide, because NMS-null mice retain circadian rhythms.	Arousal and sleep-wake regulation; nocturnal wakefulness; body-temperature rhythms; regulation of circadian period and wake time through the SIK3-HDAC4 pathway.
CCK^+^ ensembles	Heterogeneous distribution. Most CCK+ cells occupy the shell and lack direct retinohypothalamic input; discrete core-associated VIP/CCK/PK2-coexpressing subtypes have also been identified.	The predominant shell population lacks an acute response to ambient light pulses, whereas a core-associated subtype responds to acute nighttime light. CCK^+^ activity and function are strongly photoperiod dependent.	Acts as a photoperiod-dependent network switch that changes its regulation of VIP+ activity; contributes to phase control, network robustness, and adaptive plasticity.	Activity-period regulation under long photoperiod and constant light; behavioral phase shifting; modulation of jet-lag recovery.
PK2 signaling domain	PK2 expression is strongly rhythmic within the SCN and peaks during the day; PK2-neuron calcium activity peaks near midday. A CCK/PK2-coexpressing subtype has been reported.	Predominantly regulated by endogenous circadian phase rather than established as a primary sensory-input pathway. Time-of-day-dependent inhibition of PK2 signaling modifies *Per2* expression and circadian period.	Functions as a major rhythmic SCN output signal and can also feedback on neuronal firing and the TTFL in a phase-dependent manner.	Locomotor activity; sleep-wake rhythms; hormone secretion; anxiety-like behavior.

AVP, arginine vasopressin; BT, body temperature; CCK, cholecystokinin; GRP, gastrin-releasing peptide; NMS, neuromedin S; PK2, prokineticin 2; PVT/PVN, paraventricular thalamic/hypothalamic nuclei; SCN, suprachiasmatic nucleus; TTFL, transcriptional-translational feedback loop; VIP, vasoactive intestinal peptide.

### 2.1 VIP neurons: the photic gatekeepers and network synchronizers

VIP neurons are predominantly located in the ventral region of the SCN and constitute about 24% of its neuronal population (Moore et al., 2002; Abrahamson and Moore, 2001). They serve as a primary conduit for photic information, receiving direct input from the optic nerve (Tanaka et al., 1993). Their role in maintaining intra-SCN synchrony is fundamental; mice lacking VIP or its receptor VPAC2 lose coherent wheel-running rhythms under constant darkness and exhibit desynchronized electrical activity in *ex vivo* SCN slices (Vosko et al., 2007; Piggins and Cutler, 2003; Hastings et al., 2014). The restorative effect of co-culturing arrhythmic VIP-deficient SCN slices with wild-type tissue (Maywood et al., 2011), alongside the ability of daily VIP administration to induce behavioral rhythms (Maywood et al., 2011), underscores VIP's role as a coupling signal. VIP administration can phase-shift PER2::LUC molecular rhythms and the electrical activity rhythm of SCN neurons *in vitro* (An et al., 2011; Reed et al., 2001), indicating effects on both molecular and electrophysiological SCN phase. Intriguingly, while knocking out *Bmal1* specifically in VIP neurons does not significantly alter wheel-running rhythms, the genetic ablation of the VIP neurons themselves abolishes them under constant darkness (Mazuski et al., 2020; Todd et al., 2020; Shan et al., 2020). This suggests that VIP neurons can drive and synchronize network rhythmicity through mechanisms that are separable from their cell-autonomous molecular clock. Beyond circadian regulation, VIP neurons are integral to sleep regulation (Collins et al., 2020; Todd et al., 2020), with their activation via α2-adrenergic receptors promoting sleep and facilitating environmental entrainment (Zhang et al., 2024). Their function is also modulated by stress signals (Yurgel et al., 2025) and metabolic status, as a high-fat diet reduces VIP expression in the SCN (Afonso-Oramas et al., 2023). Furthermore, they are implicated in reproductive and mood regulation, as conditional knockout of *Vax1* in VIP neurons disrupts estrous cycles and increases depressive-like behaviors in female mice (Van Loh et al., 2023).

### 2.2 GRP neurons: collaborators in photic entrainment

Like VIP neurons, GRP neurons are primarily located in the SCN core and make up approximately 10% of its neurons (Abrahamson and Moore, 2001; Atkins et al., 2010; Antle and Silver, 2005). Functionally, GRP shares similarities with VIP, and some VIP neurons co-express GRP (Kawamoto et al., 2003). GRP neurons receive primarily intra-SCN projections but can also respond directly to light, as evidenced by light-induced *Fos* and *Per* gene expression and increased calcium activity (Karatsoreos et al., 2004; Bryant et al., 2000; Li et al., 2025), and both GRP and VIP can modulate clock gene expression and influence the phase of wheel-running activity (Kallingal and Mintz, 2006; Gamble et al., 2007). While GRP deficiency or targeted ablation of SCN GRP neurons does not severely compromise activity rhythms or photic entrainment (Li et al., 2025), GRP signaling contributes to network synchronization, particularly in contexts where VIP signaling is compromised, as its inhibition blocks the rescue of rhythmicity in VIP-deficient SCN co-cultures (Maywood et al., 2011). This indicates a supportive, albeit non-essential, role for GRP in maintaining network coherence under specific conditions.

Beyond circadian regulation, GRP and its receptor (GRPR) also participate in non-circadian functions, notably itch signal processing across distinct anatomical pathways. At the spinal level, GRP expressed in primary sensory dorsal root ganglion (DRG) neurons activates GRPR-expressing neurons in lamina I of the dorsal horn, which form a specific neural pathway for transmitting itch (Sun and Chen, 2007; Sun et al., 2009). In contrast, central GRP and GRPR neurons in the suprachiasmatic nucleus (SCN) operate within a subcortical visual pathway to transmit socially contagious itch. Specifically, Yu et al. (2017) first demonstrated that GRP/GRPR signaling in the SCN core mediates contagious itch transmission evoked by observing scratching conspecifics. Extending this subcortical pathway, Gao et al. (2022) revealed that ipRGCs convey visual motion cues via PACAP-PAC1R signaling to SCN GRP neurons, which then relay signals to the thalamic (PVT) and hypothalamic (PVN) paraventricular nuclei to trigger mimicry scratching and stress responses. Thus, central SCN GRP neurons belong to an ipRGC-SCN-PVT visual circuit that is functionally and anatomically segregated from classical spinal somatosensory itch pathways.

### 2.3 AVP neurons: the guardians of intrinsic rhythm and output

In contrast to the core-localized VIP and GRP neurons, AVP neurons are mainly situated in the dorsal SCN shell and comprise 10%−30% of the neuronal population (Kalsbeek et al., 2010). They exhibit robust single-cell rhythmicity, though population-level calcium signals can appear weaker due to heterogeneity (Stowie et al., 2023). AVP expression is directly activated by the BMAL1/CLOCK heterodimer and repressed by PER/CRY, placing it under direct TTFL control (Jin et al., 1999). SCN-released AVP acts as a key rhythmic output signal, inducing rhythmic electrical activity in other brain nuclei via its receptors (Tousson and Meissl, 2004). AVP neurons appear more pivotal than VIP neurons for maintaining the SCN's autonomous oscillation; knocking out *Bmal1* specifically in AVP neurons abolishes SCN rhythmicity *in vitro* (Shan et al., 2020). Similarly, AVP neuron-specific knockout of *Bmal1* or *Csnk1d* prolongs the activity period in constant darkness (Shan et al., 2020; Tsuno et al., 2023), and disrupting GABAergic neurotransmission within these neurons lengthens activity duration and impairs rhythmicity (Maejima et al., 2021). Mice lacking AVP receptors also display weakened wheel-running rhythms (Li et al., 2009a), consolidating the role of AVP neurons in regulating circadian behavior through their intrinsic clocks and neurotransmitter release. Beyond pacemaking, AVP neurons shape internal SCN networks, as their activation increases calcium activity in VIP neurons (Tsuno et al., 2023). They also exert a profound influence on physiological outputs, regulating hormone levels [e.g., restoring corticosterone to normal after SCN lesion (Kalsbeek et al., 1992)], and reproductive behavior via projections to kisspeptin neurons (Jamieson et al., 2021), though their intrinsic clock may be dispensable for fertility (Tonsfeldt et al., 2022). Mice lacking *Bmal1* specifically in AVP neurons maintain normal estrous cycles, fertility, and kisspeptin responses (Tonsfeldt et al., 2022). AVP also modulates luteinizing hormone (LH) and gonadotropin-releasing hormone (GnRH) secretion through the hypothalamic-pituitary-gonadal axis (Funabashi et al., 2000; Palm et al., 1999). Like VIP neurons, AVP neurons also respond to stress signals and co-regulate behavioral outputs with light-sensitive VIP neurons (Yurgel et al., 2025). AVP neurons also control pre-sleep drinking behavior (Gizowski et al., 2016) and modulate body temperature in response to osmotic challenges (Gizowski and Bourque, 2020), showcasing their role in integrating diverse physiological signals.

### 2.4 *Nms* expression across overlapping SCN ensembles: pacemaking and arousal

*Nms* expression does not define a neuronal population that is mutually exclusive from VIP- or AVP-expressing neurons. *Nms*-expressing cells constitute approximately 40% of mouse SCN neurons, extend across the core and shell, and encompass most but not all VIP^+^ and AVP^+^ neurons, together with additional NMS^+^/VIP^−^/AVP^−^ cells (Lee et al., 2015). Consistent with this organization, single-cell transcriptomic analyses identify *Vip*^+^/*Nms*^+^ and Avp^+^/Nms^+^ subtypes rather than a single homogeneous NMS class (Wen et al., 2020). Thus, phenotypes resulting from Nms-Cre-based genetic manipulations should be interpreted as effects of perturbing a broad and heterogeneous *Nms*-defined ensemble. Such experiments do not by themselves establish that an anatomically segregated NMS-only population, or the NMS peptide itself, mediates these functions.

Within this interpretive framework, studies targeting *Nms*-expressing cells implicate this broad ensemble in the maintenance of SCN network rhythmicity and the regulation of arousal. Disruption of cell-autonomous molecular clocks or vesicular/GABAergic transmission in *Nms*-expressing neurons markedly impairs SCN synchrony and behavioral rhythmicity under constant darkness, whereas *Nms*-null mice retain behavioral circadian rhythms (Lee et al., 2015; Bussi et al., 2023). This distinction indicates that the reported phenotypes reflect the functional importance of the heterogeneous *Nms*-defined cellular ensemble and its cotransmitters, rather than an indispensable role of the NMS peptide alone. Recent studies employing targeted neuronal ablation, optogenetics, and *in vivo* fiber photometry further show that *Nms*-expressing neurons participate in sleep–wake regulation; their disruption reduces nocturnal wakefulness and abolishes body-temperature rhythms through a feedback circuit involving the SCN, dorsomedial hypothalamus, and preoptic area (Wu et al., 2025). At the molecular level, manipulations targeting this ensemble implicate the SIK3–HDAC4 pathway in the regulation of circadian period and wake time (Asano et al., 2023). *Nms*-expressing cells also exhibit photoperiod-dependent plasticity in their abundance and projections (Porcu et al., 2022), while rhythmic changes in their primary cilia mediate Sonic Hedgehog signaling involved in SCN coupling and rhythmic-output stability (Tu et al., 2023).

### 2.5 CCK neurons: adaptive switches of the SCN network

CCK neurons in the SCN exhibit distinct cellular, anatomical, and functional heterogeneity. The vast majority of CCK neurons are localized in the SCN shell, where they lack direct synaptic input from the retinohypothalamic tract (RHT) and do not display acute electrophysiological or immediate-early gene responses to ambient light pulses (Xie et al., 2023; Hannibal et al., 2010). *In vivo* calcium imaging reveals that their activity precedes behavioral onset across various photoperiods. Ablating CCK neurons shortens the activity period and disrupts rhythms under long photoperiods and constant light, while their activation can shift activity phases and influence the speed of jet lag recovery (Xie et al., 2023). A central functional property of CCK neurons is their ability to act as adaptive switches within the SCN network. Under standard light-dark cycles, they inhibit VIP neuron activity, but under long photoperiods, they switch to promoting it. This dynamic regulation helps maintain circadian robustness and adaptability to changing environmental conditions.

In contrast to the non-retinorecipient shell population, high-resolution scRNA-seq identifies multiple CCK neuron subpopulations, including neurons co-expressing VIP and CCK-PK2 (Xu et al., 2021). Following acute nighttime light exposure, this CCK subpopulation rapidly upregulates immediate-early genes *Fos* and *Egr1* as well as NPAS4 target genes. Thus, while the predominant shell CCK population functions as a non-photic network gatekeeper, discrete core-associated CCK subpopulations actively participate in processing photic information, together orchestrating the temporal plasticity and robustness of the central circadian clock.

### 2.6 Prokineticin 2 and other signaling components

Prokineticin 2 (PK2) expression in the SCN exhibits a robust circadian rhythm, peaking during the day (Stangerup et al., 2025; Cheng et al., 2002). *In vivo* calcium imaging shows that PK2 neuron activity peaks around midday (Onodera et al., 2023), aligning with the rhythmic expression of PK2. *In vitro* studies demonstrate that inhibiting PK2 signaling lengthens the SCN clock period (Morris et al., 2021). It is widely regarded as a principal SCN output signal, as mice lacking PK2 or its receptor show weakened rhythms in locomotion, sleep-wake cycles, and hormone secretion (Li et al., 2006; Hu et al., 2007). *In vivo* administration of PK2 at night suppresses locomotor activity (Cheng et al., 2002), likely by enhancing SCN neuronal firing rates (Ren et al., 2011). PK2 deletion does not alter SCN core clock gene expression (Li et al., 2006; Prosser et al., 2007). While initially characterized as a pure output, recent evidence suggests PK2 can also influence the TTFL within the SCN, activating *Per2* expression and modulating rhythm amplitude at specific circadian phases (Morris et al., 2021). Inhibiting SCN PK2 signaling between CT20–23 suppresses *Per2* expression and lengthens the circadian period (Morris et al., 2021). Intracerebroventricular (ICV) infusion of PK2 also increases anxiety-like behavior (Li et al., 2009b), suggesting a role for PK2 neurons in mood regulation.

Beyond these major players, other signaling molecules contribute to SCN function. TGF-α, predominantly expressed in SCN glia, suppresses locomotor activity and disrupts sleep–wake cycles (Lindley et al., 2008; Kramer et al., 2001). Similarly, cardiotrophin-like cytokine (CLC) regulates activity: ICV delivery of CLC markedly reduces locomotor activity without altering the expression of core SCN clock genes, whereas blocking CLC signaling at its peak phase, either via receptor antagonists or neutralizing antibodies, elicits additional daytime activity (Kraves and Weitz, 2006). Angiotensin II (AII) and methionine-enkephalin (mENK)-expressing neurons localize to the SCN shell, and mENK colocalizes with roughly half of the vasopressin (AVP) neurons (Silver et al., 1999). Calretinin (CalR)–positive neurons reside in the ventral SCN and shell but are absent from the core, suggesting a role in SCN calcium mobilization (Moore et al., 2002; Ikeda and Allen, 2003). Neurotensin (NT) shifts the phase of SCN neuronal firing, implicating NT as an external clock-input signal (Meyer-Spasche et al., 2002; Coogan et al., 2001).

The continued refinement of SCN cellular taxonomy through scRNA-seq promises to further unravel the intricate network architecture of this central pacemaker. Park et al. (2016) defined five SCN neuronal subpopulations, *Vip, Avp, Adcyap1, Pcsk1n*, and multi-neuropeptide–expressing neurons, revealing a more intricate network architecture. Building on this, Wen et al. identified novel subtypes (*Avp*^+^/*Nms*^+^, *Grp*^+^/*Vip*^+^, *Vip*^+^/*Nms*^+^, *Cck*^+^/*C*1*ql*3^+^, and *Cck*^+^/*Bdnf*^+^ neurons), characterized their light-dependent gene expression changes across the circadian cycle, and reconstructed each subtype's 3D spatial distribution at single-cell resolution, thereby laying a solid foundation for dissecting mammalian circadian neural circuits (Wen et al., 2020).

### 2.7 From neuronal populations to network-level cue processing

These populations should be viewed as an asymmetrically coupled network rather than as independent functional modules. Retinal input preferentially recruits core VIP and GRP neurons, and VIP/GRP signaling supports network coherence and the distribution of phase information within the SCN (Maywood et al., 2011). AVP circuits stabilize ensemble phase and can increase calcium activity in VIP neurons (Yamaguchi et al., 2013; Tsuno et al., 2023), whereas CCK neurons regulate VIP activity in a photoperiod-dependent manner (Xie et al., 2023). NMS expression overlaps with VIP- and AVP-containing subpopulations (Wen et al., 2020), providing a possible link between core and shell functions, although a direct integrative role has not been demonstrated. Feeding-activated DMH RFRP/GABA projections also target VIP and GRP neurons rather than AVP neurons (Acosta-Galvan et al., 2011), identifying the retinorecipient core as a candidate site of photic-feeding convergence. Whether these signals interact within identified SCN cell types is considered in Section 3.5.

## 3 SCN encoding of temporal information

As the master circadian pacemaker, the SCN's core function is to encode, interpret, and adapt to temporal information from the environment. This process is not monolithic but varies dramatically depending on the nature, timing, and duration of the time cue. The SCN employs distinct strategies to process daily light signals, adapt to long-term seasonal changes, recover from acute disruptions like jet lag, and even integrate non-photic cues such as feeding rhythms.

### 3.1 Light stimulation: the primary zeitgeber

The SCN is anatomically biased toward photic entrainment, but the ventral core and dorsal shell should not be regarded as two autonomous modules with mutually exclusive cue preferences. Rather, the retinorecipient ventral core initiates rapid light resetting, whereas strongly rhythmic oscillators in the dorsal shell stabilize ensemble time and are rephased through reciprocal core–shell coupling. Neurons in the core, primarily VIP and GRP types, respond rapidly to photic stimulation, as evidenced by the immediate induction of *Fos* and other immediate-early genes (Colwell and Foster, 1992; Silver et al., 1996; Jac et al., 2000). This functional specialization is supported by structural plasticity; for instance, the axon branches of VIP neurons have been observed to expand during the day and retract at night, reflecting a circadian rhythm in their connectivity (Neitz et al., 2024). At the molecular level, light activation of core neurons triggers a conserved signaling cascade. Glutamate release from the RHT leads to membrane depolarization, elevating intracellular calcium and cAMP levels. This, in turn, activates CREB, which binds to cAMP response elements (CRE) in the promoter of clock genes such as *Per1* and *Per2*, ultimately shifting the phase of the molecular TTFL and consequent animal behavior (Sakamoto et al., 2013; Jagannath et al., 2013). The SCN's response to light is exquisitely time dependent, a principle formalized in the Phase Response Curve (PRC) (Ruger et al., 2013; Pittendrigh and Daan, 1976). In nocturnal rodents like mice, light exposure during the subjective day (circadian time, CT 0-12) produces minimal phase shifts, a refractory “dead zone.” In contrast, light during the early subjective night (CT 12–18) induces phase delays, while light during the late subjective night (CT 18–24) produces phase advances, thereby enabling the clock to synchronize to the external light-dark cycle.

Furthermore, SCN sensitivity is spectrally tuned. *In vitro* studies using *Per2*-luc reporter mice demonstrate that short-wavelength (blue/UV) light induces larger phase shifts in clock gene rhythms than long-wavelength (green/red) light (Buhr et al., 2015). *In vivo* electrophysiology confirms this, showing that UV light potently enhances SCN neuronal activity, whereas green light at higher irradiances can reduce sustained responses (van Diepen et al., 2021; Walmsley et al., 2015). Remarkably, even very dim monochromatic UV light (log 9.95 photons/cm^2^/s) is sufficient to activate SCN neurons (Orlowska-Feuer et al., 2020). This spectral sensitivity is translationally relevant; human studies show that morning blue light exposure improves rest-activity rhythm stability, reduces sleep fragmentation, and advances bedtime, while nighttime light exposure increases sleep latency and disrupts sleep (Esaki et al., 2016; Zerbini et al., 2020; Barroggi Constantino et al., 2025), forming the basis for light therapy in treating sleep and circadian rhythm disorders.

### 3.2 Seasonal changes: encoding day length

To survive in a changing world, organisms must adapt to seasonal fluctuations by modifying their activity patterns, migration, reproductive physiology, metabolism, and integumentary traits (Hazlerigg and Loudon, 2008). The SCN measures day length (photoperiod) and relays this seasonal information throughout the organism (Figure 2). Behaviorally, mice exhibit a compressed active phase (α) under long summer days and an expanded one under short winter days (Refinetti, 2002). The mechanism underlying this adaptation involves a network-level change within the SCN. *In vivo* electrophysiological recordings reveal that the waveform of the SCN's electrical activity rhythm changes with photoperiod, with activity onset and offset aligning with the 50% rise and fall points of the population signal, a pattern that persists for days in constant darkness, indicating a “memory” of the prior photoperiod (Houben et al., 2009). At the single-cell level, this corresponds to a change in the phase distribution of neuronal firing: under short days, SCN neurons fire in a tightly synchronized cohort around midday, whereas under long days, their firing phases become more dispersed, resulting in a broader window of elevated activity (VanderLeest et al., 2007). Single-cell analyses further confirm that SCN network coupling weakens under long photoperiods and strengthens under short ones (van Beurden et al., 2023). This photoperiodic encoding exhibits spatial specificity within the SCN. In hamsters, the phases of C-FOS and PER1 protein expression are uniform across the rostrocaudal axis under short days, but become progressively more phase-advanced in the caudal SCN relative to the mid-region (4–8 h) and rostral (8–12 h) SCN under long days (Yan and Silver, 2008). A similar phenomenon is observed in mice, where *in vitro* imaging reveals increasing phase differences in PER2 expression between the core and shell regions as day length extends (Evans et al., 2013).

**Figure 2:**
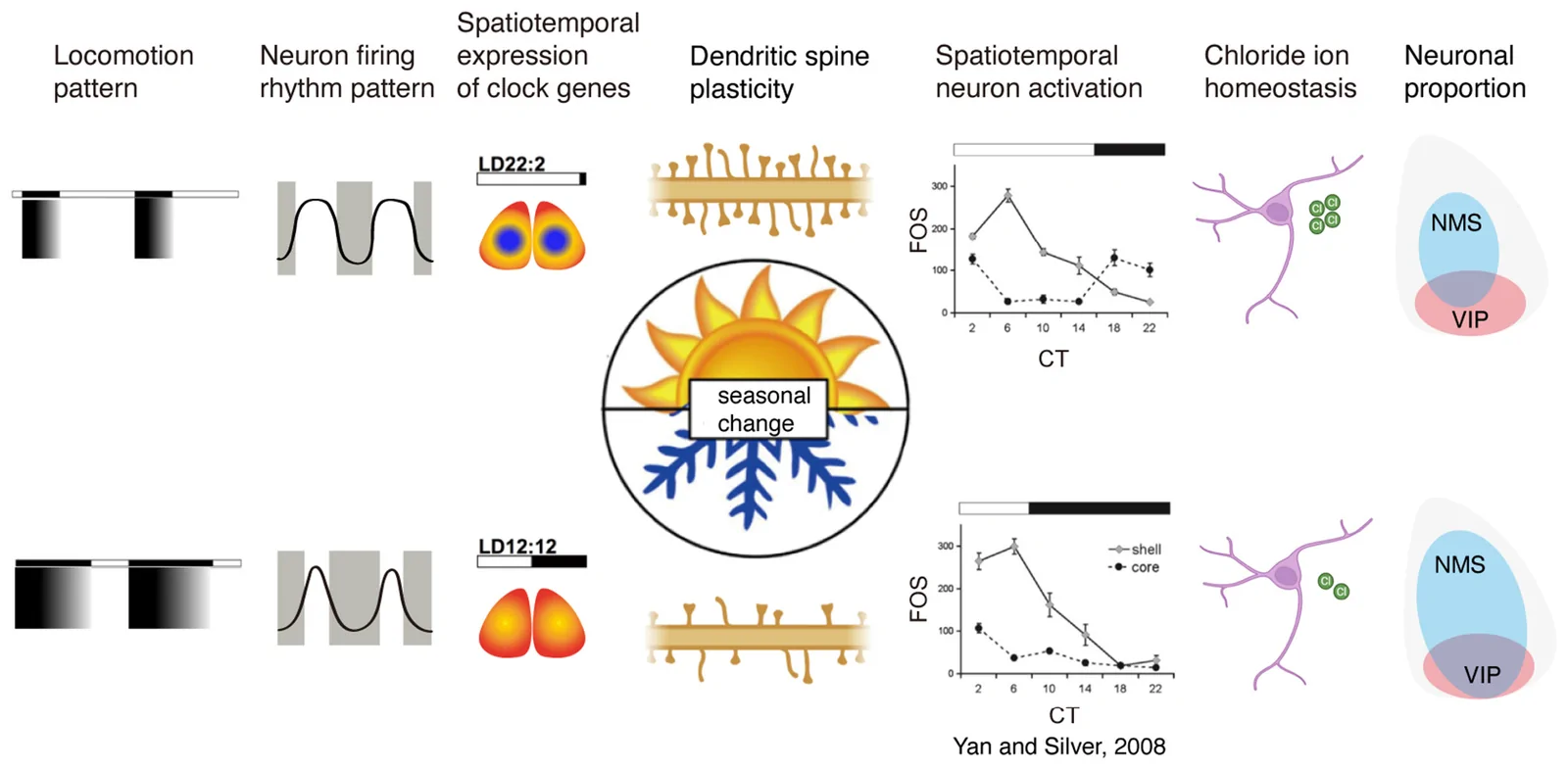
Seasonal adaptation of the SCN. The circadian clock adapts to photoperiod changes via multiple mechanisms, including locomotion, neuronal electrical activity, spatial expression patterns of clock genes in the SCN, dendritic spine plasticity, spatial patterns of SCN neuronal activation, neuronal chloride homeostasis, and the proportions of different neuron types.

At the molecular level, these changes are underpinned by alterations in ion channel expression. A key mechanism involves a photoperiod-driven shift in the balance between the chloride importer NKCC1 (promoting chloride influx) and the exporter KCC2 (facilitating chloride efflux). This leads to higher intracellular chloride concentrations under long days, which transforms the effect of the neurotransmitter GABA from inhibitory to excitatory, thereby altering network dynamics and signal propagation (Myung et al., 2015; Farajnia et al., 2016, 2014). Seasonal adaptation also involves structural plasticity, with changes in the number and projections of neuropeptide-specific neurons like NMS and VIP, and mediation by microRNAs such as miR-132/212 (Porcu et al., 2022; Mendoza-Viveros et al., 2017). Although most studies of SCN photic encoding emphasize neuronal physiology and molecular pathways, emerging high-resolution imaging and electron microscopy techniques offer promising tools to uncover finer-scale structural adaptations.

### 3.3 Jet lag: internal desynchronization and recovery

Rapid travel across time zones creates a mismatch between the internal circadian clock and the external environment, leading to the malaise known as jet lag. This state arises from the desynchronization among different tissue clocks, which reentrain at different rates (Nagano et al., 2003). The recovery process begins with internal desynchrony within the SCN itself. Following an abrupt shift in the light-dark cycle, the phase of the neuronal activity rhythm in the light-sensitive ventral SCN adjusts rapidly, whereas the corresponding rhythm in the dorsal shell shifts more slowly, producing transient regional desynchrony (Albus et al., 2005). This pattern is mirrored at the molecular level, where the resetting of clock gene expression (e.g., *Per1, Per2*, and *Cry1*) occurs faster in the ventral SCN than in the dorsal SCN (Nagano et al., 2003; Davidson et al., 2009). In contrast, peripheral tissue clocks generally reentrain more slowly than the SCN in response to a new schedule (Yamazaki et al., 2000), explaining the prolonged physiological discomfort during jet lag recovery.

### 3.4 SCN response to dietary signals

#### 3.4.1 SCN response to time-restricted feeding (TRF)

Time-restricted feeding (TRF), which confines food access to a fixed daily window without reducing caloric intake, is a powerful entrainer for peripheral clocks. In mice, daytime TRF inverts the phase of clock gene expression in several peripheral tissues including the liver, kidney, heart, and pancreas but exerts minimal impact on SCN core clock gene expression (Damiola et al., 2000; Stokkan et al., 2001; Mukherji et al., 2015). These effects can occur independently of the SCN master clock (Hara et al., 2001). Several lines of evidence support the SCN's resistance to TRF: 3 weeks of TRF did not alter C-FOS expression in the SCN (Angeles-Castellanos et al., 2004); 2-h TRF restricted to ZT4–ZT6 failed to affect PER2 expression in the rat SCN (Natsubori et al., 2013); and 1 month of TRF under LD or LL conditions had no effect on SCN neuronal electrical activity recorded from single neurons (Shibata et al., 1983). Notably, TRF can restore hepatic gene expression rhythms in clock-deficient mice (Vollmers et al., 2009), demonstrating the existence of SCN-independent entrainment pathways.

However, a growing body of evidence challenges the dogma of SCN immunity to feeding cues, revealing conditions under which dietary signals can modulate the central pacemaker. The effect of TRF on the SCN is dependent on genetic background. Under constant darkness (DD), 4–6 months of daytime 6-h TRF is sufficient to entrain wheel-running activity and PER2 expression in the suprachiasmatic nucleus (SCN) in a subset of house mouse strains (Castillo et al., 2004). After 3–4 weeks of 3-h TRF under DD, CS mice exhibited wheel-running activity phases aligned with the previous TRF window upon return to *ad libitum* feeding, along with corresponding phases in SCN clock gene expression (Abe et al., 2007). In contrast, C57BL/6J (B6) mice displayed free-running activity, and SCN gene expression matched their intrinsic circadian rhythm (Abe et al., 2007). Diet composition and palatability also influence these variables. In rats, providing a palatable chocolate bar daily for 6 weeks can shift wheel-running activity and alter SCN expression of C-FOS and PER1 proteins (Mendoza et al., 2005a). Similarly, high-fat TRF, timed caloric restriction, or hypocaloric feeding can significantly modify the expression of SCN output signals like AVP and induce substantial phase advances in activity rhythms (Kalsbeek et al., 1998; Caldelas et al., 2005; Andrade et al., 2004; Mendoza et al., 2007, 2005b).

Feeding-related effects on the SCN are strongly dependent on the phase and experimental context of the intervention. Independent studies have shown that scheduled feeding can entrain or shift SCN molecular rhythms in selected mouse strains or sensitized genetic backgrounds (Castillo et al., 2004; Abe et al., 2007; Bouchard-Cannon and Cheng, 2012), that timed hypocaloric or palatable feeding can modify SCN clock-gene, immediate-early-gene, and neuropeptide expression (Caldelas et al., 2005; Mendoza et al., 2005a,b, 2007), and that TRF can restore SCN PER2 rhythmicity when the network is rendered arrhythmic by constant light (Lamont et al., 2005). Within this broader literature, Zhai reported that ZT0–ZT4 TRF altered Ca^2+^ activity in SCN GABAergic neurons and produced a persistent locomotor aftereffect (Zhai et al., 2022). This finding provides network-level evidence for a phase-dependent SCN response to feeding schedules, but it does not by itself establish universal resetting of the SCN TTFL or identify the SCN as the exclusive encoder of feeding time. Indeed, feeding-time representations also arise in extra-SCN circuits; under TRF, AgRP-neuron activity remained synchronized to the feeding window after SCN ablation and in constant darkness, with prodynorphin-expressing DMH neurons contributing to this adaptation (Sayar-Atasoy et al., 2024).

Taken together, these findings support a hierarchy of feeding-related SCN responses rather than uniform resetting of the central clock. To logically reconcile these seemingly paradoxical findings and address the mechanism of SCN feeding sensitivity, it is essential to distinguish between resetting the core cell-autonomous transcriptional-translational feedback loop (TTFL) in SCN clock neurons and modulating neuronal firing rates, calcium dynamics, or systemic output signals. At the core TTFL level, feeding cues primarily regulate the expression phase of clock genes and proteins such as *Per1, Per2*, and *Bmal1*. Resetting the core SCN TTFL occurs only under defined permissive conditions, such as specific genetic susceptibility (Castillo et al., 2004; Abe et al., 2007), or compromised SCN network states induced by LL (Lamont et al., 2005). At the level of neuronal firing and calcium dynamics, the available evidence is protocol-dependent. Changes at this level should therefore be interpreted as network responsiveness unless accompanied by a persistent phase shift of core TTFL markers under constant conditions. At the level of systemic output signals, metabolic cues can uncouple downstream SCN outputs from the core clockwork. Dietary interventions, such as high-fat TRF, timed caloric restriction, hypocaloric feeding, or palatable food, can alter SCN C-FOS expression and downregulate output neuropeptides like AVP, thereby inducing substantial phase advances in behavioral activity and physiological rhythms even when the core SCN TTFL remains anchored to the light-dark cycle (Kalsbeek et al., 1998; Andrade et al., 2004; Angeles-Castellanos et al., 2004; Mendoza et al., 2005a,b, 2007; Caldelas et al., 2005). Thus, feeding sensitivity is well-supported, but resetting of the SCN molecular clock is conditional rather than routine.

#### 3.4.2 SCN modulation of an extra-SCN food-anticipatory activity (FAA) system

When food access is restricted to a fixed daily interval, rodents develop increased locomotor and food-seeking activity approximately 1–3 h before mealtime, termed food anticipatory activity (FAA). Importantly, FAA persists after surgical SCN lesions and in animals with severe genetic disruption of SCN identity or clock function (Stephan et al., 1979a,b; Wilcox et al., 2021). Food-anticipatory behavior is also retained in *Per1*/*Per2*/*Per3* triple-knockout mice lacking a functional canonical SCN clock (Ehichioya et al., 2023). These findings demonstrate that an intact SCN is not required for FAA and argue against the SCN acting as the autonomous oscillator that generates this behavior. Instead, the timing mechanism underlying FAA is generally attributed to one or more extra-SCN food-entrainable oscillators embedded within a distributed network. The DMH, cerebellum, and food-responsive peripheral oscillators have each been implicated as candidate or contributing components, although no single site has been established as the necessary and sufficient food-entrainable oscillator (Landry et al., 2006, 2007; Mendoza et al., 2010; LeSauter et al., 2009).

The persistence of FAA without a functional SCN does not, however, exclude modulation by SCN-associated circuits. Feeding-activated RFRP/GABA neurons in the DMH project to the retinorecipient ventral SCN, and interactions between the DMH and SCN can influence the intensity of anticipatory activity (Acosta-Galvan et al., 2011). Similarly, the NPY-expressing IGL–SCN pathway contributes to FAA, as acute inhibition of IGL terminals in the SCN reduces its behavioral expression (Fernandez et al., 2020). Collectively, these findings indicate that the SCN may gate or coordinate the magnitude and temporal expression of FAA within the light-entrained circadian framework, rather than directly and autonomously generating the underlying food-anticipatory rhythm.

#### 3.4.3 Astrocytes as potential targets for dietary signals

Astrocytes, which constitute about one-third of all glial cells, are now recognized as active partners in SCN timekeeping and potential mediators of dietary effects. They are key regulators of synaptic function, energy metabolism, and gliotransmitter release, influencing feeding, cognition, and circadian rhythms (Sofroniew and Vinters, 2010; González-García et al., 2021). Tso et al. demonstrated that astrocytes synchronize the SCN clock. Using CRISPR/Cas9 to knock out *Bmal1* specifically in SCN astrocytes prolonged *Per2* expression rhythms and lengthened the wheel-running period. Similarly, knockout of *Csnk1d* in SCN astrocytes extended SCN clock gene rhythms and behavioral activity cycles (Tso et al., 2017; Brancaccio et al., 2017). Astrocytes exhibit rhythmic calcium levels (peaking at night in antiphase to neuronal activity) and release glutamate rhythmically, which can inhibit SCN neuronal firing through dorsally located NMDAR2C-expressing neurons (Brancaccio et al., 2017). Knocking out *Bmal1* in SCN glial cells also alters activity rhythms and impairs cognitive function (Barca-Mayo et al., 2017). The rhythmic release of GABA by astrocytes also modulates SCN excitability. Extracellular GABA levels in the SCN oscillate in antiphase to neuronal activity (Patton et al., 2023). Pharmacological disruption of astrocytic GABA release desynchronizes SCN neuronal calcium rhythms and clock gene expression *in vitro* (Ness et al., 2025). Restoring the TTFL in glial cells reestablishes extracellular GABA rhythms and rescues SCN rhythmicity in clock-deficient tissue (Patton et al., 2023), indicating that astrocytes function upstream in regulating circadian output. Multi-omics analysis has further identified specific molecular interactions, such as the ANXA2–S100A10 complex in astrocytes, that enable SCN network coordination (Patton et al., 2025).

SCN astrocytes are established regulators of local circadian timekeeping through intrinsic molecular clocks and rhythmic glutamatergic and GABAergic signaling (Tso et al., 2017; Brancaccio et al., 2017; Patton et al., 2023; Ness et al., 2025). However, whether they directly detect circulating feeding-related hormones or nutrients remains unresolved. Evidence that hypothalamic astrocytes respond to leptin and insulin through LepR and InsR, respectively, and express the glucose transporter GLUT2 derives primarily from the arcuate nucleus (ARC), median eminence (ME), and mediobasal hypothalamus (Young and McKenzie, 2004; Cheunsuang and Morris, 2005; Kim et al., 2014; García-Cáceres et al., 2016; González-García et al., 2021; Murat and García-Cáceres, 2021). The ME and adjacent ARC possess specialized vascular interfaces that facilitate exposure to circulating signals, whereas the entry of leptin into other brain regions is regulated by the blood–brain and blood–CSF barriers (Cheunsuang and Morris, 2005; Di Spiezio et al., 2018). Comparable access and cell-autonomous, receptor-dependent responses have not, to our knowledge, been demonstrated for SCN astrocytes. Astrocyte-wide deletion of the insulin receptor affects circadian behavior (González-Vila et al., 2023), but this manipulation is not SCN-specific and therefore does not establish a direct blood-to-SCN-astrocyte pathway. Nutritional state can nevertheless affect SCN glia: high-salt feeding engages astrocyte-enriched endothelin-B receptor signaling and increases nighttime SCN neuronal excitability (Paul et al., 2025), whereas chronic starvation is associated with reduced SCN glial density (Zimmermann et al., 2025). These findings support modulation of SCN glia by nutritional state without demonstrating LepR-, InsR-, or GLUT2-dependent direct sensing. We therefore present direct metabolic sensing by SCN astrocytes as a working hypothesis. Feeding-related information may instead, or additionally, reach the SCN through RFRP/GABAergic DMH projections to the retinorecipient ventral SCN and NPY-positive IGL projections (Acosta-Galvan et al., 2011; Saderi et al., 2013; Fernandez et al., 2020).

Based on these findings, we present an integrative working model of environmental influences on the SCN (Figure 3). The model distinguishes established pathways from candidate routes. Direct retinal input to retinorecipient VIP/GRP neurons through the RHT and intrinsic astrocytic clock and gliotransmitter functions are well supported. DMH and IGL projections provide supported neural routes through which feeding-related information may influence the SCN, although their cell-type-specific targets within the SCN remain incompletely defined. By contrast, direct sensing of circulating insulin, leptin, or glucose by SCN astrocytes remains a working hypothesis. Thus, the model does not imply that all feeding-related signals converge through a single established SCN circuit.

**Figure 3:**
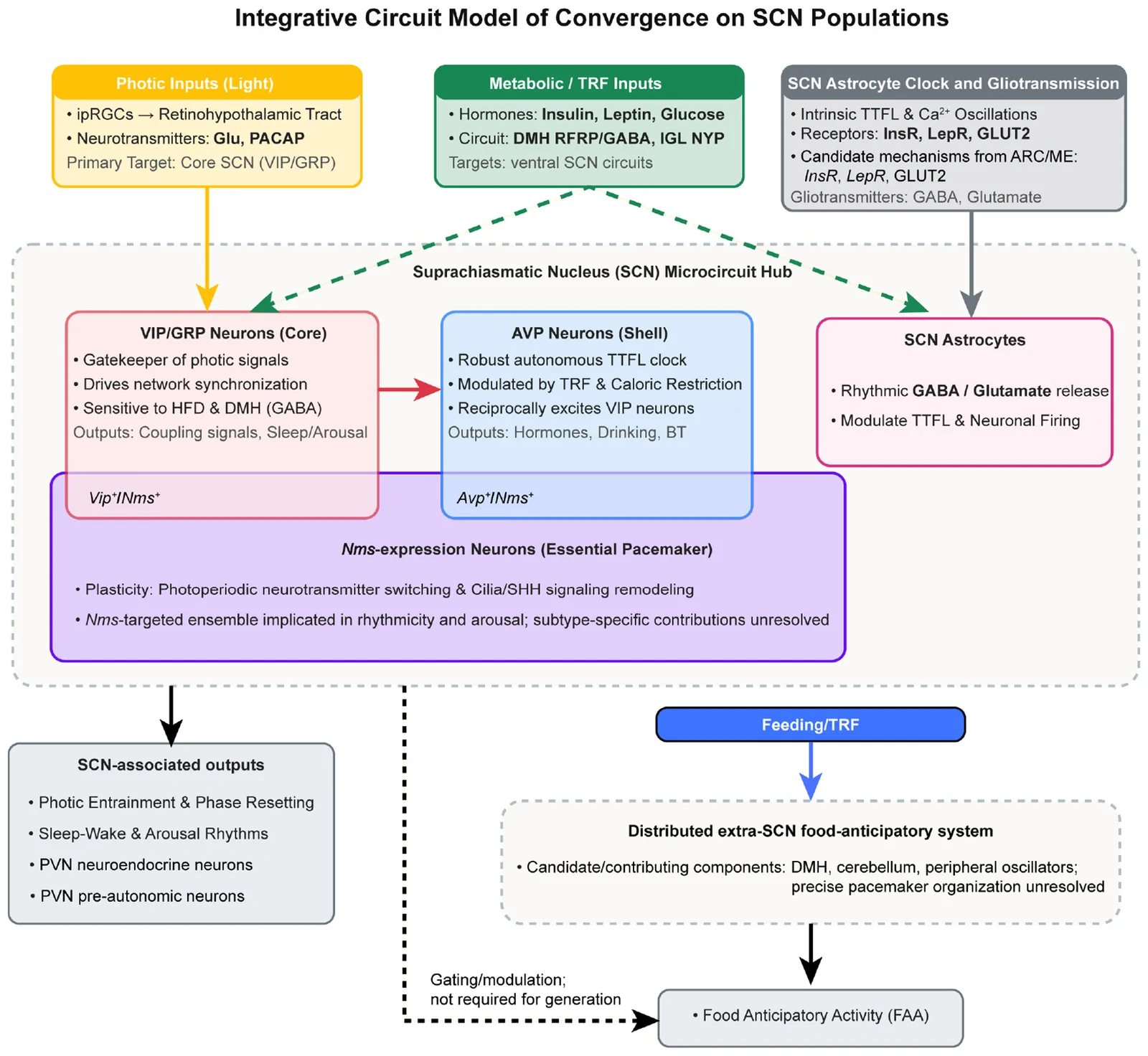
SCN integration of environmental signals and modulation of extra-SCN food anticipation. Photic signals from ipRGCs reach VIP/GRP neurons in the SCN core through the retinohypothalamic tract, whereas feeding-related signals may influence SCN circuits through the DMH and IGL pathways, circulating factors, and astrocytes. Intrinsic astrocytic clocks and rhythmic GABA/glutamate signaling are established regulators of SCN excitability, whereas direct sensing of feeding-related insulin, leptin, or glucose by SCN astrocytes remains a proposed mechanism. NMS is represented as an overlapping marker within subsets of VIP^+^ and AVP^+^ neurons rather than as an independent downstream population, and the subtype-specific roles of *Nms*-expressing neurons in integrating photic and metabolic inputs remain unresolved. The timing drive for FAA is depicted separately as arising from a distributed extra-SCN food-anticipatory system, for which the DMH, cerebellum, and peripheral oscillators are candidate or contributing components rather than a single established locus. The dashed SCN–FAA arrow denotes gating or modulation of FAA expression and does not imply autonomous generation by the SCN. Solid arrows indicate established primary pathways or outputs; dashed arrows indicate modulatory or proposed relationships. SCN efferents also engage PVN neuroendocrine and branch-selective pre-autonomic neurons, which relay circadian timing to peripheral metabolic organs through hormonal, sympathetic, and parasympathetic pathways.

### 3.5 Conflicting photic and feeding cues

When the phase information carried by light and feeding diverges, the SCN does not appear to resolve the conflict through an instantaneous, unilateral dominance of one signal. Instead, the mismatch is initially expressed as spatial and systemic desynchrony and is subsequently reconciled through network coupling. The classical core and shell framework is therefore better understood as an asymmetrically coupled architecture with differential access to afferent signals and distinct resetting dynamics, rather than as a strict division between a photic core and a metabolic shell. Following an abrupt advance of the light-dark cycle, *Per1, Per2*, and *Cry1* rhythms reset rapidly in the ventrolateral SCN but require several days to shift in the dorsomedial SCN, resulting in transient intranuclear desynchrony (Kawamoto et al., 2003; Nagano et al., 2003). GABAergic signaling conveys phase information between these regional oscillators (Albus et al., 2005), whereas AVP signaling through V1a and V1b receptors confers network stability and resistance to rapid phase perturbation (Yamaguchi et al., 2013). Thus, photic resetting begins preferentially in the ventral core, but coherent reentrainment requires the new phase to propagate through the dorsal shell.

In addition, feeding-related information is not confined to the dorsal shell. Feeding anticipation activates RFRP/GABA neurons in the dorsomedial hypothalamus that project specifically to the retinorecipient ventral SCN, targeting VIP and GRP neurons rather than AVP neurons (Acosta-Galvan et al., 2011). In parallel, the IGL-NPY pathway conveys metabolic and feeding-related information to the SCN and contributes to food anticipatory activity (Saderi et al., 2013; Fernandez et al., 2020). These anatomical findings indicate that photic and feeding-related signals can access common circuits within the ventral SCN. Importantly, this convergence should not be interpreted as localizing the food-entrainable oscillator (FEO), or the primary timing mechanism underlying FAA, within the SCN; rather, it provides a potential route through which the SCN may gate or modulate the behavioral expression of FAA, whose timing is attributed primarily to distributed extra-SCN circuits. Nevertheless, under a stable light-dark cycle and normocaloric daytime feeding, the SCN molecular clock generally remains aligned with light, whereas peripheral clocks follow meal timing (Damiola et al., 2000; Mukherji et al., 2015). A common outcome of cue conflict is therefore partitioned entrainment: central SCN phase remains predominantly light-locked, while liver and other peripheral oscillators become food-entrained, producing internal circadian misalignment.

Partitioned entrainment should not be taken to imply that a light-locked SCN is disconnected from peripheral metabolism. The SCN conveys circadian phase downstream through neuroendocrine and autonomic routes centered on the paraventricular nucleus of the hypothalamus (PVN). Viral-tracing studies have identified largely segregated pre-sympathetic and pre-parasympathetic neuronal populations in the PVN, with corresponding branch-selective organization extending upstream to the SCN (Buijs et al., 2003). PVN pre-sympathetic neurons relay to sympathetic preganglionic neurons in the spinal intermediolateral column, whereas pre-parasympathetic neurons engage brainstem vagal circuits, thereby coordinating rhythmic functions of the liver, pancreas, adrenal gland, and other metabolic organs. VIP-containing SCN axons innervate a subset of spinal-projecting PVN neurons (Teclemariam-Mesbah et al., 1997), although the transmitter logic is not exclusively peptidergic: SCN GABAergic and glutamatergic inputs to the PVN exert causal control over sympathetic hepatic glucose production (Kalsbeek et al., 2004, 2008). In parallel, SCN-derived VIP- and AVP-associated signals regulate PVN neuroendocrine activity and glucocorticoid secretion, providing a systemic hormonal timing cue (Kalsbeek et al., 1992; Jones et al., 2021). Thus, during photic–feeding conflict, peripheral tissues may simultaneously receive meal-derived entraining signals and SCN-timed autonomic and endocrine outputs, providing a mechanistic basis for internal metabolic misalignment.

The relative weighting of these cues is, however, state-dependent. Daytime hypocaloric feeding, but not normocaloric feeding at the same phase, advances the rhythms of selected SCN clock genes and AVP expression and modifies both the behavioral phase-response curve and light-induced *Per1* expression (Mendoza et al., 2005b). Thus, caloric deficit, palatability, feeding phase, intervention duration, genetic background, and the integrity of the SCN network can all increase the influence of feeding-related signals. Combined jet-lag and feeding paradigms further demonstrate this phase dependence. Following a 6-h advance of the light-dark cycle, scheduled feeding aligned with the new night accelerates the reentrainment of behavioral and extra-SCN oscillations (Ubaldo-Reyes et al., 2017). Conversely, during chronically alternating light-dark schedules, food availability fixed to the original phase delays adaptation when it falls within the new light phase, whereas feeding restricted to the current dark phase accelerates the adaptation of activity and body temperature rhythms (Schilperoort et al., 2019).

Region-resolved *c-Fos* analyses provide an additional mechanistic clue. After a 6-h light-dark phase advance, ventrolateral SCN activation remained strongly associated with light onset, whereas a palatable meal delivered at the onset of the new night restored the day-night c-FOS rhythm in the dorsomedial SCN. The same meal cue delivered at the previous night onset failed to restore this dorsal rhythm and impeded behavioral reentrainment (Escobar et al., 2020). Although *c-Fos* is a marker of neuronal activation rather than a direct measure of clock phase, these findings suggest that feeding aligned with the new photic phase can reinforce phase propagation toward dorsal and output-related SCN compartments, whereas feeding aligned with the previous phase prolongs competition between the two temporal signals.

Taken together, the available combined-cue studies demonstrate state-dependent interaction at regional and behavioral levels, but they do not yet define a cell-type-resolved integration mechanism. Direct evidence is strongest for co-access of retinal and feeding-related DMH pathways to VIP/GRP-containing ventral circuits, together with AVP-dependent stabilization of core-shell phase relationships. In contrast, how identified VIP, GRP, AVP, NMS, and CCK neurons change their firing, calcium dynamics, or molecular phase when light and feeding cues are presented simultaneously remains largely unknown. How simultaneous photic and feeding cues reshape SCN-to-PVN autonomic and neuroendocrine outputs also remains an important unresolved question.

### 3.6 Biological sex as a modulator of SCN circuit organization and function

Biological sex should be considered as a source of heterogeneity in SCN organization. Under normal conditions, women show higher melatonin amplitude but lower core body temperature amplitude than men, with both rhythms occurring earlier relative to sleep, illustrating that sex effects are output-specific (Cain et al., 2010). The circuit substrate is also sexually dimorphic. In the human SCN, the rostrocaudal extent of the AVP subdivision is greater in women, whereas the number of VIP immunoreactive neurons shows an age-dependent sex difference: young men have approximately twice as many VIP neurons as young women, the direction reverses in middle age, and the difference disappears in older adults (Hofman et al., 1996). In mice, Erα- and Erβ-expressing SCN cells show sex and gonadal hormone-dependent distributions, while androgen receptors are enriched in the retinorecipient ventrolateral core and are expressed more strongly in males (Karatsoreos et al., 2007). These findings suggest that biological sex may influence clock function at multiple levels, potentially including photic input processing, core and shell coupling, and peptidergic output.

Research shows that output-level metrics can differ even in the presence of stable baseline molecular oscillations or uniform responses between sexes. In C57BL/6J mice, daytime firing rates in dorsal SCN neurons are higher in males than in females and this difference is abolished by GABA type A receptor blockade, indicating sex-dependent GABAergic tone. The same study found more precise activity onset in males, longer activity duration in females under constant darkness, and larger light-induced phase shifts in gonadally intact females than in intact males, but no sex difference in the phase or amplitude of SCN PER2::LUC rhythms *ex vivo*; by contrast, liver and adrenal PER2::LUC amplitudes were lower in females (Kuljis et al., 2013). Cell-type-specific recordings provide a more direct correlate of amplitude: male rat SCN AVP neurons display higher acrophase firing, a greater circadian firing amplitude, and a slightly later peak than female AVP neurons, consistent with the larger amplitude of cerebrospinal fluid AVP rhythms reported in males (Michaud et al., 2023). Moreover, deletion of *Vax1* from VIP neurons shortens behavioral and *ex vivo* SCN period in both sexes, yet produces sex-specific reproductive, endocrine, and affective outcomes (Van Loh et al., 2023).

Gonadal steroids actively tune the coupling and resilience of this circuit. In SCN explants, 17β-estradiol acting through ERβ increases connexin 36 and connexin 43 expression and stabilizes circadian amplitude when AVP receptor signaling is inhibited; blocking these gap junction proteins reduces amplitude, suggesting that estrogen reinforces coupling between neurons and astrocytes (Schlaeger et al., 2024). *In vivo*, ovariectomy without estradiol replacement reduces rhythm power and locomotor activity, increases vulnerability to arrhythmicity in constant light, and attenuates early-night photic phase delays, whereas estradiol replacement restores these responses (Michaud et al., 2023). Conversely, gonadectomy in males lengthens the free-running period, reduces activity onset precision, and alters SCN synaptic and glial markers and photic responses; testosterone or non-aromatizable dihydrotestosterone reverses these effects (Karatsoreos et al., 2007; Iwahana et al., 2008; Karatsoreos et al., 2011). Estrous state also modifies adaptation to shifted lighting. Proestrous females reentrain behavioral and peripheral rhythms faster than metestrous females after a 6-h phase advance, although their SCN clock does not reset equivalently faster (Pilorz et al., 2020).

### 3.7 Aging and SCN responsiveness to non-photic cues

Aging does not uniformly abolish SCN responsiveness to non-photic cues. Instead, it alters both the fidelity of afferent signals reaching the SCN and the network that converts those signals into a coherent phase adjustment. Two principal routes for behavioral non-photic information are the geniculohypothalamic tract, which supplies NPY from the IGL, and serotonergic projections from the midbrain raphe. In rats, aging causes a selective loss of NPY-immunoreactive IGL neurons while sparing GAD and enkephalin immunoreactivity in IGL, which could reduce the capacity of this pathway to convey behavioral and metabolic timing information to the SCN (Fiuza et al., 2016). In Syrian hamsters, aging also increases SCN 5-HT1B receptor binding at ZT6, and this age-dependent change is prevented by chronic fluoxetine, showing that serotonergic modulation within the SCN is remodeled with age (Duncan et al., 2010).

This distinction is supported by direct SCN studies. In SCN slices from approximately 4- and 15-month-old mice, NPY produced comparably robust phase advances of the neuronal firing-rate rhythm, even though responses to several other phase-resetting agents were impaired with age (Biello, 2009). Thus, at least one electrophysiological phase-response pathway remains functional into middle age. Network recruitment is nevertheless an additional likely constraint. The aged mouse SCN contains more neurons that peak out of phase with the dominant rhythm and exhibits reduced-amplitude GABAergic postsynaptic currents (Farajnia et al., 2012). In addition, old SCN slices were shown to have a greater proportion of excitatory GABA responses, an increased excitation to inhibition ratio, and elevated basal intracellular Ca^2+^ levels, changes that can modify the phase adjustment and coupling processes through which a local input is propagated across the clock network (Olde Engberink et al., 2023). These findings imply that impaired afferent NPY/5-HT signaling and altered intercellular coupling diminish the aged SCN's ability to filter transient behavioral or metabolic noise from genuine non-photic zeitgebers.

Exercise and meal timing can influence the aged SCN under an intact light-dark cycle, but the available evidence does not uniformly establish entrainment of the core TTFL. In aged mice, access to a running wheel increased the amplitude of the *ex vivo* SCN firing-rate rhythm and partially improved the persistence of SCN PER2::LUC rhythmicity after an advance of the light-dark cycle (Leise et al., 2013). Daytime TRF also produced age-dependent changes in the daily profiles of serotonin and its metabolites in the SCN of 3-, 12-, and 24-month-old rats (Reddy et al., 2022). These findings demonstrate residual electrophysiological, molecular, and neurochemical responsiveness, but the TRF study in particular does not directly demonstrate feeding-induced resetting of the aged SCN molecular clock.

### 3.8 Circadian medicine: non-pharmacological and pharmacological strategies

Circadian medicine treats biological time as a modifiable component of disease prevention and therapy rather than as a background variable. It can be organized into three complementary strategies: training the clock through environmental and behavioral interventions, clocking the drug by administering established therapies at an appropriate circadian phase, and drugging the clock by pharmacologically modifying the circadian system itself (Sulli et al., 2018; Cederroth et al., 2019). The SCN occupies a central position in this framework because it receives retinal light information and coordinates sleep, endocrine, autonomic, and body temperature rhythms that synchronize peripheral tissues. However, the SCN is not the sole therapeutic target, because feeding, exercise and many pharmacological agents can also act directly on extra-SCN and peripheral oscillators. The goal of circadian medicine should therefore be to restore alignment among the SCN, behavior and organ-specific clocks rather than shifting one component in isolation.

Among non-pharmacological interventions, timed light exposure provides the most direct means of resetting the SCN through the retinohypothalamic tract. Because the direction and magnitude of light-induced resetting follow a phase-response curve, the same light stimulus can advance or delay the circadian system depending on when it is delivered. Light therapy should therefore consider circadian phase, spectrum, intensity and duration, together with strategic reduction of evening and nocturnal light (Khalsa et al., 2003; Auger et al., 2015). Regular sleep-wake timing and appropriately scheduled periods of darkness can further stabilize the light-dark and rest-activity cycles reaching the SCN. Timed exercise represents an additional non-photic intervention. Human phase response curves based on melatonin metabolites demonstrate that exercise can induce phase advances or delays according to its timing, while exercise also directly influences clocks and metabolic pathways in skeletal muscle and other peripheral tissues (Youngstedt et al., 2019; Gabriel and Zierath, 2019). Consequently, exercise should not be prescribed as universally optimal in the morning or evening. Its timing should instead be selected according to the desired phase shift, individual chronotype and metabolic or performance objective.

Building on the mechanisms discussed above, time-restricted feeding, termed time-restricted eating in human studies, provides a complementary approach that predominantly targets peripheral clocks. In a controlled human study, delaying three daily meals by 5 h delayed the plasma glucose rhythm by approximately 5.7 h and adipose tissue *PER2* expression by approximately 1 h, without changing dim light melatonin onset or the cortisol rhythm, which are commonly used markers of central circadian timing (Wehrens et al., 2017). This finding illustrates how meal timing can alter the phase relationship between SCN-driven and peripheral rhythms without necessarily resetting the SCN. Early time-restricted eating improved insulin sensitivity, blood pressure, and several other cardiometabolic measures in men with prediabetes, whereas a later eating window from 12:00 to 20:00 did not produce greater weight loss than a consistent meal schedule in adults with overweight or obesity (Sutton et al., 2018; Lowe et al., 2020). Although these trials differed in design and population, they indicate that the biological timing of the eating window, caloric intake, adherence and clinical endpoint can all influence efficacy (Manoogian et al., 2022). Thus, an eating window aligned with the intended active phase and separated from the biological night is more biologically rational than applying a fixed clock-time window to all individuals.

Pharmacological circadian medicine includes both chronobiotics and the circadian scheduling of conventional drugs. Melatonin is produced under SCN control and feeds back onto the SCN, where MT1 receptor activation inhibits neuronal firing and MT2 receptor activation contributes to phase shifting of circadian activity (Dubocovich, 2007). Appropriately timed melatonin and melatonin receptor agonists can therefore facilitate entrainment, although their phase-shifting effects depend on administration relative to endogenous circadian phase rather than simply on administration at bedtime. The MT1/MT2 agonist tasimelteon, for example, entrained circadian rhythms in a proportion of totally blind individuals with non-24-h sleep-wake disorder, providing clinical evidence that pharmacological manipulation of melatonin signaling can entrain the human central pacemaker (Lockley et al., 2015). A second strategy, chronotherapy, schedules an existing drug to coincide with rhythms in disease activity, drug targets, absorption, metabolism or clearance, thereby potentially increasing efficacy or reducing toxicity (Dallmann et al., 2016). Nighttime modified-release prednisone, designed to release the drug before the early-morning inflammatory peak, reduced morning stiffness in rheumatoid arthritis (Buttgereit et al., 2008). However, treatment timing cannot be generalized across an entire drug class. The large TIME trial found no difference in major cardiovascular outcomes between morning and evening administration of usual antihypertensive medications (Mackenzie et al., 2022). These findings emphasize that chronotherapy should be drug-specific, disease-specific and endpoint-specific rather than based on a universal recommendation for morning or bedtime dosing.

A more direct pharmacological strategy is to target the molecular clock or coupling mechanisms within the SCN network. Small molecules have been developed against CRYs, casein kinase 1, REV-ERBs, RORs and other clock regulators (Chen et al., 2018). For example, KL001 stabilizes CRY proteins and lengthens the circadian period, whereas nobiletin enhances circadian amplitude through ROR-associated mechanisms and improves metabolic phenotypes in mouse models (Hirota et al., 2012; He et al., 2016). At the SCN network level, pharmacological blockade of V1a and V1b vasopressin receptors reduces interneuronal resistance to phase perturbation and accelerates reentrainment after a shifted light-dark cycle in mice (Yamaguchi et al., 2013). These findings suggest that both the intracellular oscillator and SCN intercellular coupling can be pharmacologically modified. Nevertheless, most direct clock modulators and SCN coupling agents remain preclinical. Their translation will require evaluation of tissue specificity, off-target effects, long-term safety and the possibility that shifting the SCN or a peripheral clock alone could increase internal desynchronization.

### 3.9 Temperature compensation and conditional thermal responsiveness of the SCN

The central SCN clock is notably temperature-compensated. Temperature compensation should not be equated with temperature insensitivity. In circadian biology, temperature compensation refers specifically to the maintenance of a near 24 h free-running period across different sustained temperatures, rather than the absence of acute thermal responses. In rat SCN slices, the periods of neuronal firing rhythms were 23.9 h at 37 °C and 23.7 h at 31 °C, yielding a Q_10_ value of 0.99 and demonstrating compensation of circadian period (Ruby et al., 1999). The intact adult SCN network is also substantially less susceptible than peripheral oscillators to entrainment by physiologically relevant temperature cycles, partly because intercellular coupling stabilizes the collective oscillator against thermal perturbations (Buhr et al., 2010; Abraham et al., 2010).

Recent studies further define the limits of this resistance. In cultured SCN neurons, transcriptional and intracellular Ca^2+^ rhythms were maintained at 22 to 28 °C but suspended at approximately 15 °C. Rewarming reset the Ca^2+^ rhythm and subsequently restored its phase relationship with transcriptional oscillations, indicating that moderate hypothermia can be compensated whereas deep hypothermia crosses a functional boundary of the SCN clock (Enoki et al., 2023). Temperature changes can also regulate SCN-controlled pathways without resetting the core oscillator. Cooling altered the expression and circadian periodicity of the cold-response gene *Rbm3*, whereas the period of *Per2* remained largely unaffected. This response required an intact BMAL1 complement, suggesting that BMAL1 links temperature sensing to downstream clock-controlled transcription while preserving core clock timing (Hoekstra et al., 2024). Consistent with an important contribution from organism-level regulation, a recent *in vivo* study found that SCN neuronal firing rhythms and peripheral PER2::LUC rhythms persisted with largely preserved periods during a Q neuron-induced hypothermic state of approximately 25 °C, although their amplitudes were reduced. By contrast, SCN and kidney slices maintained at the same temperature showed strongly attenuated or reset oscillations (Hirano et al., 2026).

Systemic thermal stress should therefore be distinguished from experimentally imposed local warming of the SCN. Whole-body heat exposure recruits peripheral thermoafferent pathways and hypothalamic thermoregulatory circuits, particularly warm-sensitive neurons in the preoptic area. TRPC4-expressing GABAergic warm-sensitive neurons detect internal warming and contribute to heat-defense and fever responses (Zhou et al., 2023). Prolonged heat exposure additionally induces intrinsic warm sensitivity and neuronal plasticity in preoptic LepR-expressing neurons, thereby promoting systemic heat tolerance (Ambroziak et al., 2025). Activation of these pathways may modify autonomic, endocrine, metabolic, and arousal signals associated with the SCN, but it does not by itself demonstrate direct resetting or loss of temperature compensation in the SCN core oscillator. These results suggest that SCN thermal resistance is best understood as preservation of circadian period during modest physiological temperature fluctuations.

### 3.10 Translational relevance of multi-cue circadian misalignment: shift work, social jetlag, and metabolic syndrome

Shift work and social jetlag represent common human conditions in which photic and non-photic time cues remain chronically or recurrently misaligned. Night work combines light exposure, wakefulness, physical activity, and food intake during the biological night with daytime sleep and frequently irregular fasting and feeding. Because the light-responsive SCN and oscillators responsive to behavioral and metabolic cues differ in their sensitivity and resetting kinetics, adaptation is often incomplete. In police officers working rotating shifts, only a minority showed melatonin or cortisol adaptation toward a night-oriented circadian phase after night shifts (Kosmadopoulos et al., 2024). Likewise, simulated night work caused a loss of temporal coordination between the peripheral blood mononuclear cell transcriptome and the shifted behavioral cycle (Kervezee et al., 2018). The DMH and IGL pathways described above provide plausible neural routes through which feeding and behavioral timing may influence the SCN, although their contribution to human adaptation remains inferential. Because direct recording of the human SCN is not feasible, dim-light melatonin onset and core body temperature should be interpreted as markers of central circadian phase rather than direct measurements of SCN neuronal state. Shift work is therefore better conceptualized as partitioned entrainment among the central pacemaker, behavior, and peripheral tissues than as uniform failure of the SCN.

Controlled human experiments demonstrate that this internal misalignment has immediate metabolic consequences. Forced-desynchrony and simulated night-work protocols decreased leptin, increased postprandial glucose and insulin, and impaired glucose tolerance independently of the effects of circadian phase or the behavioral cycle (Scheer et al., 2009; Morris et al., 2015). Consistent with these experimental findings, night shift work, particularly rotating schedules that include frequent night shifts, was associated with higher odds of type 2 diabetes in the UK Biobank (Vetter et al., 2018). Meal timing provides particularly relevant evidence for the contribution of non-photic signals. During simulated night work, restricting food intake to the daytime prevented misalignment between central circadian phase and the endogenous glucose rhythm and prevented the deterioration of glucose tolerance observed when participants ate during both day and night (Chellappa et al., 2021). A related analysis further showed that daytime eating attenuated adverse changes in cardiac autonomic regulation and the prothrombotic marker PAI-1 (Chellappa et al., 2025). Thus, appropriately timing a non-photic cue can reduce physiological disruption even when the sleep-wake schedule remains misaligned.

Social jetlag represents a milder but recurrent form of cue conflict and is commonly defined as the difference in sleep midpoint between work and free days (Wittmann et al., 2006). These shifts in sleep timing are frequently accompanied by changes in light exposure, activity, and meal timing, repeatedly altering both photic and non-photic inputs before stable reentrainment can occur. In the New Hoorn Study, social jetlag exceeding 2 h was associated with a higher prevalence of metabolic syndrome and diabetes or prediabetes, particularly among participants younger than 61 years (Koopman et al., 2017). In adolescents, delayed and irregular sleep timing and social jetlag greater than 1.5 h strengthened the association between visceral adiposity and metabolic syndrome severity (Morales-Ghinaglia et al., 2024). Together, these observations suggest that recurrent misalignment among light, sleep, activity, and feeding can disturb glucose regulation, autonomic function, adiposity, and blood pressure, thereby contributing to the pathophysiological cluster underlying metabolic syndrome. However, social jetlag is a behavioral proxy rather than a direct measure of SCN phase, and most available evidence remains observational. Future studies should therefore combine dim-light melatonin onset, actigraphy, meal timing, continuous glucose monitoring, and peripheral molecular rhythms to distinguish central circadian misalignment from sleep loss and peripheral desynchronization.

## 4 Discussion

After half a century of intensive research, the suprachiasmatic nucleus (SCN) is firmly established as the central circadian pacemaker of the mammalian brain. The SCN generates and coordinates ~24-h rhythms through cell-autonomous TTFLs and a hierarchically organized network that synchronizes peripheral physiology. However, the prevailing narrative of the SCN has undergone a profound shift. It is no longer adequately described as a mere rigid metronome, faithfully but inflexibly tracking the solar day. Instead, a new consensus is emerging that recasts the SCN as a sophisticated and dynamic coordinator, a compact neural circuit that actively weighs, prioritizes, and adapts to a complex stream of environmental time cues, from the dominant photic signals to potent non-photic inputs like feeding rhythms and metabolic status.

This integrative capacity is hardwired into the SCN's remarkable cellular heterogeneity. Mass spectrometry and scRNA-seq have unveiled a vast neuropeptidergic landscape and functionally specialized neuronal subtypes. Viewed at the network level, these cell populations are not parallel functional modules. VIP/GRP core neurons provide privileged access for retinal and some feeding-related signals, AVP circuits stabilize and propagate ensemble phase, and CCK and NMS populations may adjust coupling, plasticity, and output state. Current evidence therefore supports an asymmetrically coupled architecture with state-dependent cue weighting. However, because most studies manipulate one zeitgeber at a time, functional integration by identified neuronal populations remains a testable hypothesis rather than a settled mechanism. Beyond this neuronal architecture, astrocytes have emerged as active partners, contributing their own circadian clocks and serving as potential metabolic sensors that may translate dietary signals into changes in gliotransmission and network function. Together, neuronal interactions and astrocytic signaling make temporal encoding a network-level property. A revelation of recent years is the demonstrable plasticity of the SCN network in response to feeding cues. While the SCN maintains a gated hierarchy with light as the primary zeitgeber, evidence shows that under specific conditions of genetic background, diet composition, and especially feeding timing, time-restricted feeding (TRF) can significantly modulate SCN neuronal activity, gene expression, and behavioral output. Accordingly, SCN sensitivity to feeding should not be equated with molecular-clock resetting. In most intact animals under a stable light-dark cycle, TRF modifies network activity or output while the core TTFL remains predominantly light-locked; direct molecular entrainment has been observed only under defined strain, lighting, feeding, or network conditions. In addition, temperature provides an additional boundary condition for SCN plasticity. Intercellular coupling protects ensemble clock phase from routine physiological temperature fluctuations, whereas sufficiently strong, phase-specific, or systemic thermal challenges can modify SCN phase, temperature-responsive outputs, or thermoregulatory memory without necessarily producing equivalent resetting of the core TTFL.

Based on this refined understanding and the current frontiers of the field, we propose several prioritized and interconnected avenues for future research that will help unlock the next chapter in circadian biology:

### 4.1 From cellular taxonomy to circuit-level integration

Future work must bridge the gap between identifying cell types and understanding their real-time functional interactions. A key goal is to systematically investigate the roles of less-characterized SCN neuropeptides (e.g., NT, mENK, and AII) and the receptor-mediated pathways through which they influence the TTFL and downstream targets. More importantly, research should focus on elucidating the molecular and cellular mechanisms by which key neuronal subtypes (VIP, AVP, and GRP) integrate multiple inputs, such as concurrent light and feeding signals, at both the single-cell and network levels. Demonstrating functional integration will require simultaneous photic and feeding manipulations, cell-type-resolved recordings, and pathway-specific perturbations showing that one cue modifies the response to the other; anatomical co-access alone demonstrates potential convergence rather than integration. Employing emerging circuit-mapping tools, such as transsynaptic viral tracing combined with targeted pathway ablation or activation *in vivo*, will help validate and dissect the SCN's input-output logic.

At minimum, convincing evidence would require a factorial design comparing no-cue, light-only, feeding-only, and combined-cue conditions across aligned and conflicting phases; a statistically supported photic-by-feeding interaction rather than the sum of independent responses; cell-type-resolved recordings; and pathway-specific perturbation showing that the interaction depends on defined circuit elements. Claims should be restricted to the measured level, and any claim of integration at the molecular-clock level should additionally require a persistent change in rhythmic TTFL phase under constant conditions or after cue withdrawal.

### 4.2 Defining non-canonical SCN functions in physiology

The research focus is decisively shifting beyond the SCN's role in regulating core rhythm outputs (e.g., locomotor activity). A major frontier involves investigating the roles of marker-defined and partially overlapping neuronal ensembles (VIP, AVP, NMS, and CCK) in controlling a wider range of physiological processes, including stress responses, fluid balance, reproduction, and metabolism. Understanding how the SCN integrates these homeostatic signals to modulate its own output and, in turn, regulate systemic physiology will provide a more holistic view of its function in health and disease.

### 4.3 Resolving the controversy: a systematic approach to TRF and SCN plasticity

The debate surrounding the SCN's sensitivity to feeding cues necessitates a more systematic and controlled experimental approach. Future studies must be designed to comprehensively evaluate how different TRF paradigms, systematically varying critical parameters such as mouse strain, diet type (e.g., high-fat vs. chow), feeding phase (across the entire circadian cycle), duration, and intervention length, affect SCN neuronal activity, clock gene expression, neuropeptide release, and rhythmic output. Particular focus should be placed on identifying the critical windows (e.g., the transition between activity and rest phases) where feeding signals are most effective in inducing SCN plasticity, and on investigating the long-term (months) consequences of TRF on SCN homeostasis and environmental responsiveness.

### 4.4 Elucidating the role of astrocytes as metabolic sentinels

A pivotal and relatively unexplored area is the mechanism by which SCN astrocytes detect dietary and energy status. Research should investigate how hormones and metabolites fluctuating with TRF (e.g., insulin, leptin, and glucose) are sensed by astrocytic receptors (*InsR, LepR*) and transporters (GLUTs). It will be crucial to elucidate how these diet-driven alterations in astrocyte metabolism and intracellular signaling translate into changes in the rhythmic release of gliotransmitters (glutamate, GABA, and ATP), and how this gliotransmission ultimately modulates neighboring SCN neuronal excitability, network coupling, and the TTFL to shape behavior. Developing and utilizing astrocyte-specific manipulation tools will validate their role as mediators of dietary effects on the central clock.

### 4.5 Probing structural and network plasticity with advanced technologies

The study of SCN plasticity must extend beyond molecular and electrical activity to encompass structural adaptations. As pioneering work by Wang et al. (2024) has shown, SCN neurons generate rhythmic calcium signals across multiple timescales, encoding rich temporal information. Future studies should employ high-resolution *in vivo* imaging, multimodal recordings (calcium, EEG, and locomotion), electron microscopy, and spatial transcriptomics to investigate adaptive changes in SCN axon dynamics, dendritic spine morphology, synaptic structure and function, and neuron-astrocyte interfaces under environmental challenges such as photoperiod shifts, jet lag, and TRF. Focus should center on identifying the molecular mechanisms, such as microRNAs and cytoskeletal regulators, that, govern this neuronal morphological plasticity and its interplay with known ion channel and neurotransmitter alterations.

Finally, this deep knowledge must be translated into practical applications. We must optimize light therapy strategies for jet lag, seasonal affective disorder, and shift work by leveraging the SCN's wavelength sensitivity and PRC properties to develop personalized, timed light exposure protocols. Concurrently, we should investigate how TRF timing can be harnessed to modulate SCN plasticity to accelerate jet lag recovery or improve circadian-related metabolic disorders. Furthermore, the continued identification of key neuropeptide pathways and astrocytic functions opens the door for developing novel rhythm-modulating drugs.

In conclusion, the suprachiasmatic nucleus stands as a brilliant exemplar of biological complexity, where a compact nucleus solves the formidable problem of environmental timekeeping not through rigidity, but through cellular specialization, dynamic network integration, and remarkable plasticity. Embracing this dynamic and integrative view will support pioneering the next generation of circadian medicine, opening new avenues for preventing and treating a wide spectrum of chronic diseases.
